# Effectiveness of interprofessional education by on-field training for medical students, with a pre-post design

**DOI:** 10.1186/s12909-015-0409-z

**Published:** 2015-07-29

**Authors:** Renzo Zanotti, Giada Sartor, Cristina Canova

**Affiliations:** 1 Department of Molecular Medicine, University of Padova, Via Loredan 18, 35131 Padova, Italy; 2 Department of Pediatrics, Hospital of Treviso, PiazzaleOspedale 1, 31100 Treviso, Italy

**Keywords:** Interprofessional education, Interdisciplinary education perception scale, Interprofessional training, Medical students

## Abstract

**Background:**

Interprofessional Education (IPE) implies how to achieve successful teamwork, and is based on collaborative practice which enhance occasions for relationships between two or more healthcare professions. This study evaluates the effectiveness of IPE in changing attitudes after a training recently introduced to medical education for second-year students at the University of Padova, Italy.

**Methods:**

All medical students following a new program for IPE were enrolled in this study. The Interdisciplinary Education Perception Scale (IEPS) was administered before and after training, according to observation-based and practice-based learning. Data were analysed with Student's paired t-test and Wilcoxon's signed rank test.

**Results:**

277 medical students completed both questionnaires. Statistically significant improvements were found in students' overall attitudes as measured by the IEPS and four subscale scores. Gender-stratified analyses showed that improvements were observed only in female students in subscale 4 (“Understanding Others’ Values”). Students who had a physician and/or health worker in their family did not show any improvement in subscales 2 (“Perceived need for cooperation”) or 4 (“Understanding Others’ Values”).

**Conclusions:**

Our results indicate that IPE training has a positive influence on students’ understanding of collaboration and better attitudes in interprofessional teamwork. More research is needed to explore other factors which may influence specific perceptions among medical students.

**Electronic supplementary material:**

The online version of this article (doi:10.1186/s12909-015-0409-z) contains supplementary material, which is available to authorized users.

## Background

Interprofessionality and good teamwork, on which interprofessional collaboration is founded, have proved to be important factors in clinical settings - to the extent that, if these kinds of collaboration are lacking, patient outcomes are negatively affected, leading to decreased work satisfaction on the part of professionals and waste of resources [1]. Interprofessional collaboration (IPC) has been defined as “multiple health workers from different professional backgrounds work together with patients, families, carers and communities to deliver the highest quality of care. It allows health workers to engage any individual whose skills can help achieve local health goals” [2]. Several studies have shown that IPC improves patient safety, the skills of each healthcare team member, and healthcare services in general [3, 4]. By extension, the efficiency and quality of care may also depend on the degree of IPC among health workers [5].

IPC requires regular education, to promote the required skills and competences for effective interprofessional team work [4]. Interprofessional Education (IPE) may be one key to promote the competences needed for efficient IPC [6] and to reduce the barriers and preconceptions existing among various healthcare groups [7]. Interprofessional education , has been recently defined by the WHO as “occasions when two or more professionals learn with, from and about each other to improve collaboration and the quality of care” [2]. However, this definition appears to focus too much on relational aspects in practice, neglecting other important dimensions such as the integration of active educational methods in the social dimensions of health organisation and daily routine.

In some European countries such as the UK, IPE development has been promoted by the government as a means for improving collaboration between health and social care professionals [8] and since 1997 at least two-thirds of UK universities with qualifying courses in health have included a pre-qualifying IPE in their core curricula [9]. In 1996, Linköping University in Sweden implemented an extensive commitment to interprofessional education for all health science students [2]. Other formalised IPE programs were set up in Canada [10]. In Italy, only recently IPE programs have been included in the core curricula of medicine or healthcare degrees in some universities, although these programs are still local, without any nation-wide formalisation.

The literature on IPE is of increasing interest as regards to the effectiveness of educational strategies of such programs such programs [11]. Some authors suggest that it should be taught during pre-qualification, as an investment in future professional practice and patient care [12]; others suggest introducing it both before and after qualification [13]. Barr (2005) proposed a five-point classification of IPE strategies to facilitate learning, which is now taken as a point of reference in the literature: problem-based, exchange-based, simulation-based, observation-based and practice-based learning (cited by 7, p74). One of the most frequently used instrument to assess student attitudes before, during and after IPE programs is the Interdisciplinary Education Perception Scale (IEPS) [14], which aims at measuring one or more IPE outcomes based on the framework of Barr [15]. Pre-qualification IPE programs have been extensively evaluated with the IEPS scale in studies on several groups of healthcare students and involving strategies such as practice-based learning [4, 16–18], problem-based learning [8, 19, 20] or simulation-based learning [10]. Most interprofessional education programs reported in the literature focus on student to student interactions from a range of health professions [10, 16, 17, 19, 20]. Although some studies have included medical students [8, 10, 17, 19, 21], none of them has tailored an IPE course aimed specifically for medical students only.

At the University of Padova, following several years of discussions, in 2013 a new observation- and practice-based IPE program specifically designed for medical students was set up. It was not only based on on-field structured observations about relationships between health care professionals, but also introduced mandatory clinical activities and operational skills whereby medical students learn from and about other professions through interactions with qualified health professionals in the following areas: working environment, knowledge of own professional competence, interprofessional relationships, and relationships with patients. As this is an innovative IPE program, knowledge of its effectiveness is lacking.

This study, using a pre-post design and the IEPS as the instrument for evaluation, aimed at measuring whether attitudes towards interprofessional teamwork among medical students improved after the introduction of the new training program and if such improvements were homogeneous as regards students’ individual characteristics.

## Methods

### Participants and study design

The target population consisted of all 421 s-year medical students enrolled in the mandatory course “Interactions with Healthcare Professions”, which required 10 h of theory in class and 40 h of training in interprofessional clinical settings over one or two weeks. Theory and training classes were designed as two programs, both mandatory, carrying two evaluations. Information and instructions about the organization of the training program were provided at the end of the theory classes.

All students who attended the course were sent emails asking them to take part in the study, together with information about the course, contact details for further explanations or technical problems, and a direct link to the on-line questionnaire.

In order to assess IPE training (described in detail below) effectiveness in developing interprofessionality, a pre-post design was adopted to evaluate students’ interdisciplinary perception before and after training. All students were asked to complete the IEPS questionnaire (see below for details) before selecting their preferred unit, and again at the end of the course. The training program started in February 2013 and data collection was completed in December 2013, the project having taken 11 months in all.

The study was approved by the Ethics Committee of the University of Padova School of Medicine.

### Training in Interprofessional Education (IPE) in a clinical setting

Experimental training in IPE in a clinical setting was based on two main educational strategies: i) on-field observations based on grounded theory approach [22], and ii) skilled activities, tutored by nurses or other healthcare professionals. The first educational strategy, on-field observation, was based on personal interactions and use of observational grids, derived from the literature adopted in the theoretical course. In that course, aspects such as gender, role, age, local traditions, cultural expectations and stereotypes were critically discussed and compared with the selected articles and students’ personal experiences and opinions. Students were asked to use these grids in clinical setting to focus their attention on relationships within professional teams and to compare their observations with results from the relevant literature provided to them, as part of the provided “training package”. Observed variables in the grids included: main characteristics of the working environment, individuals’ perception of own and others competence, and various communication behaviors connoting the relationships with various health professionals and with patients. The second educational strategy involved a review of students' experience of various technical and healthcare activities, such as taking blood and other biological samples, examining and recording health readings, providing support for walking, personal hygiene and some minor therapeutic measures. Students were involved in these activities in an interactive way with various health professionals, including nurses, nursing assistants, physioherapists, radiologists, and physicians. Such participated experiences were tutored by nurses or other healthcare professionals, preferably non-physician clinical staff. At the end of the clinical setting period, students were asked to provide a semi-structured report in which they gave details about their experience, evaluated their learning, and made comments on the quality of the observed relationships between professionals and with patients, based on the grid variables. Students were also asked to complete the report with critical comparisons between their observations and the literature provided. However, this was considered optional, in order to stimulate students without exceeding in formal requirements after the clinical experience. Clinical educational training was carried out in hospital wards in the Veneto Region (NE Italy), mostly in Padova and its surroundings.

Students were asked to indicate (by e-mail) where they preferred to be placed for training, choosing one location from a list of available settings. Selected locations were fed back to a control desk by a relational database specifically designed for managing allocations and pairing tutors with students. Information about matched students/settings were then sent both to the selected hospital wards and the students who preferred them. Students received in advance copies of the training manual, which gave information on goals, rules and other requirements, and a facsimile of the final report, to be completed one week after the end of training.

### Study instrument and data collection

The study instrument comprised two questionnaires. The first collected personal information on students (only before IPE training) such as age, gender, previous study in the healthcare field, previous work experience or voluntary work in it, presence of family members working in healthcare, and information on any previous hospitalisations. The second consisted of the IEPS developed by Luecht [23] to assess students' attitudes towards interprofessional cooperation (both before and after IPE training). The questionnaire had been translated into Italian by two professional translators, working independently. The two versions were compared and any differences were discussed and agreed upon by a group of professional healthcare experts. The IEPS has been extensively used in studies among medical, nursing and other healthcare groups of students, and has shown itself to have good reliability and validity [23]. It is composed of 18 items measured on a 5- or 6-point Likert scale. We adopted the 5-point scale, ranging from "strongly disagree" ([1] point) to "strongly agree" (5 points), as in other studies [17, 24]. Higher scores indicate a more positive attitude towards interprofessional education.

Each of the 18 items was classified into four subscales, identified and labelled according to Luecht [23], adding up to the values of the individual items (reported in Additional file 1) of the corresponding factor. Subscale 1, labelled by Luecht as “Competency and Autonomy” (items 1, 3, 4, 5, 7, 9, 10 and 13; minimum score: 8; maximum score: 40), measures how highly students respect their profession, in the sense that it is well taught and contributes significantly to improving the healthcare field, and to what extent they believe that other professions are respected in a similar fashion. Subscale 2, “Perceived Need for Cooperation” (items 6 and 8; minimum score: 2; maximum score: 10), reflects students' perceptions of the need for teamwork which typically respects and works well with other professions. Subscale 3, “Perception of Actual Cooperation” (items 2, 14, 15, 16 and 17; minimum score: 5; maximum score: 25), reveals students’ perception that their profession typically respects and works well with other professions. Subscale 4, “Understanding Others’ Values” (items 11, 12 and 18; minimum score: 3; maximum score: 15), reflects the degree of respect for contributions from all healthcare professions [23, 25].

### Data analysis

The open source application "Limesurvey 2.00” was used to ask students to take part in the survey by e-mail, and to collect and save data before and after training. The program assigned each student an anonymous ID, used to fill in and later to link the pre- and post-online questionnaires. We analysed data regarding students who attended IPE training and completed both questionnaires, before and after training. χ2 and Student’s t-test for independent samples were used to compare the gender and age distributions of the students included and not included in analyses.

A descriptive analysis was carried out to identify students' characteristics and their frequency of answers to the IEPS 18-item pre-post training scale. Since the IEPS items are ordinal in nature, Wilcoxon's signed rank test was used to analyse each item. To evaluate internal consistency of the overall IEPS and of each subscale scores (pre-training), Cronbach’s alphas were calculated. The means of the overall IEPS score and the four subscales were evaluated with Student's two-tailed paired t-test for continuous measures, to detect any differences before and after training. The assumption of normality for each subscale was evaluated with the Kolmogorov-Smirnov test. Analyses were subsequently stratified by students’ individual characteristics as gender, previous training in medical field, work or voluntary experience in the healthcare field, having a family member working in healthcare and previous history of hospitalisation. The significance level was set at 0.05. All analyses were performed with Stata SE 11 (Stata software version 11; Stata Corp., College Station, TX).

## Results

A total of 421 students participated in training, and 277 (65 %) completed all the questions before and after IPE training and were therefore included in the study. Of the 144 students not included, 28 could not be considered because, for personal reasons, they had trained before the beginning of data collection; 47 students did not complete the pre-test, and 69 did not complete the post-test. The 277 students included in the study had similar age and gender distributions to the 116 out of the 144 students with personal data available who were not included in the analyses (*p* > 0.05; data not shown). The average age of respondents was 21.18 years; 54.15 % were women and 45.85 % men (Table 1).

**Table 1:** Characteristics of study population (*n* = 277)

Characteristics	Mean (SD)	Frequency (%)
Age	21.18(0.93)	
Gender		
Female		150 (54.15)
Male		127 (45.85)
Healthcare work experience^a^ (yes)		12 (4.33)
Healthcare education plus University^b^ (yes)		27 (9.75)
Physician in family (yes)		60 (21.66)
Healthcare worker in family (yes)		74 (26.71)
Healthcare voluntary work experience (yes)		49 (17.69)
Any hospitalisation (yes)		89 (32.13)

^a^defined as previous work in healthcare field (e.g., nursing, physiotherapy, obstetrics, biology, etc.)

^b^defined as previous training in medical field (e.g., nursing, biology, obstetrics, pharmacy, short first aid courses, psychology, etc.)

Frequencies for each of the IEPS pre-post training items are described in Additional file 1. Wilcoxon's signed rank test analyses indicated a statistically significant improvement (*p* < 0.05) after IPE training for each item. The instrument showed a high overall reliability, with a Cronbach’s alpha of 0.84, although the reliability of the subscales ranged from 0.26 (subscale 4), 0.84 (subscale 3) (subscale 2: 0.55, subscale1: 0.74) consistently with previous results [17, 23].

Student's paired t-test was used to analyse pre-post training differences in the four subscales. As shown in Table 2, all four scores showed statistically significant improvements after IPE training. Subscales 1 (“Competency and Autonomy”) and 3 (“Perception of Actual Cooperation”) showed improvements of 7 % and 10 %; subscales 2 (“Perceived Need for Cooperation”) and 4 (“Understanding Others’ Values”) increased less, by 4 % and 3 %, respectively. This clinical IPE training therefore led to significant improvements in students’ perceptions of interprofessional practice, which may lead to more collaborative attitudes as future doctors.

**Table 2:** IEPS mean subscale scores of medical students' pre-post training (*n* = 277)

Subscale	Pre: mean (SD)	Post: mean (SD)	*P*-value^a^
1. Competency & Autonomy	29.23 (3.51)	31.17 (3.58)	<0.001
2. Perceived need for cooperation	8.52 (1.21)	8.89 (1.12)	<0.001
3. Perception of actual cooperation	17.67 (2.92)	19.43 (2.90)	<0.001
4. Understanding others' values	9.99 (1.62)	10.30 (1.65)	0.005

^a^Student's paired t-test

Gender-stratified analysis showed an improvement after IPE training for the first three subscales in both men and women. Subscale 4 (“Understanding Others’ Values”) improved only among women (Table 3).

**Table 3:** IEP mean subscale scores of medical students' pre-post training (*n* = 277), stratified by gender

Subscale	Males			Females		
	pre: mean (SD)	post: mean (SD)	*P*-value^a^	pre: mean (SD)	post: mean (SD)	*P*-value^a^
1. Competency & Autonomy	29.35(0.31)	31.17 (0.34)	<0.001	29.13 (0.29)	31.16 (0.28)	<0.001
2. Perceived need for cooperation	8.27 (0.12)	8.75 (0.10)	<0.001	8.73 (0.09)	9.01 (0.09)	<0.001
3. Perception of actual cooperation	17.50 (0.26)	19.28 (0.27)	<0.001	17.81 (0.24)	19.55 (0.22)	<0.001
4. Understanding others' values	9.90 (0.14)	10.13 (0.15)	0.069	10.06 (0.14)	10.43 (0.13)	0.004

^a^Student's paired t-test

The 100 students with a family member working in healthcare, compared with the 177 students without such figures, showed improvements after training only on subscales 1 and 3. No significant change after the training was found for subscales 2 and 4, in the group of students with doctors or healthcare workers in the family (*p* > 0.05) (Table 4). Other student features, such as previous training in medical field, work or voluntary experience in the healthcare field, and previous history of hospitalisation did not modify improvements in attitudes after the IPE training.

**Table 4:** IEPS mean subscale scores of medical students' pre-post training (*n* = 277), stratified by students with family members working in healthcare

Subscale	Physician or health worker in family (*n* = 100)			No physician or health worker in family (*n* = 177)		
	pre: mean (SD)	post: mean (SD)	*P*-value^a^	pre: mean (SD)	post: mean (SD)	*P*-value^a^
1. Competency & Autonomy	29.06 (3.89)	31.06 (3.79)	<0.001	29.15 (3.28)	31.23 (3.46)	<0.001
2. Perceived need for cooperation	8.49 (1.14)	8.68 (1.20)	0.081	8.53 (1.26)	9.01 (1.05)	<0.001
3. Perception of actual cooperation	18.05 (3.16)	19.07 (3.10)	<0.001	17.45 (2.76)	19.63 (2.77)	<0.001
4. Understanding others' values	10.02 (1.82)	10.05 (1.87)	0.860	9.95 (1.46)	10.45 (1.53)	<0.001

^a^Student's paired t-test

## Discussion

Interprofessional education has proved to be a challenge, requiring effective and educational strategies based on teamwork in clinical settings in which students must be directly and actively involved. A properly structured program based on on-field training with a mix of guided observations, shared interprofessional experiences and coaching, is expected to improve attitudes towards teamwork to a greater extent than traditional, less active/interactive programs. A new program of training in IPE, to complete the traditional short academic theoretical course for second-year medical students, was therefore developed at the University of Padova. To the best of our knowledge, this is the first study assessing the effect of a specific training program on interprofessional skills in medical students enrolled in an Italian university.

The structured program adopted for IPE training was based on two educational strategies: a) on-field observations, consistent with the grounded theory method [25], with the aim of discovering the basic social processes underlying teamwork, and b) structured experience in some skilled care activities performed daily and supervised by nurses or other health professionals. The basic idea was to have the medical students tutored by nurses or other professionals in clinical activities, and exposed to the skills of others. In the meantime, students were asked to observe and record interprofessional relationships from the prospective young students not yet involved in a professional role.

This combined use of the two strategies in IPE training for medical students has not been reported in other similar studies. Our study succeeded in demonstrating improved attitudes towards interprofessional collaboration among medical students more effectively than other studies evaluating IPE effectiveness in the same type of students with the IEPS [10, 17, 19, 26]. However, the above studies adopted different IPE strategies involving many types of healthcare students (nursing, medical, physiotherapy, occupational therapy, etc.). Using problem-based learning with real patients, Goelen (2006) presented five interdisciplinary seminars to undergraduate medical, nursing and physiotherapy students. Their results showed that it was only among students of medicine that no significant improvements in attitude were revealed, whereas all the others showed significant changes [19]. Other studies have evaluated students' attitudes only after the IPE experience, reporting differences among professions and, again, lower scores among medical students [10, 17, 26].

Results from quantitative observations collected pre-post training showed significant improvements in students' attitudes towards interprofessionality in all four subscales. These improvements were particularly notable in the subscales “Perceived autonomy competence within the profession” and “Perception of actual co-operation between their profession and those of others”. Such positive results are of particular interest, since this is the first study assessing the effect of an IPE university program on the attitudes of Italian medical students. The least increase was observed in subscales 2 (“Perceived Need for Cooperation”) and 4 (“Understanding Others’ Values”). Similarly, Neill [16] using the IEPS in a pre-post study based on collaborative learning in a rural community on a sample of 114 students in various healthcare professions, did not find any pre-post increase in scores on the subscale “Perceived Need for Cooperation”. Giordano [17] administered the IEPS after an interdisciplinary course taught to a sample of 496 students in the medical and healthcare professions, and found that subscale 4 scored lowest for students in all disciplines.

Our data were analysed according to the model of Luecht [23], subsequently modified by McFayden [27] in an attempt to increase its sensitivity to undergraduates. The latter author suggested that the original subscale 4 is acceptable for use among more mature undergraduates who have already had experience of clinical placements, post-graduate students with clinical experience, or practitioners, because the three original items of subscale 4 (“Understanding Others' Values”) may not be appropriate for assessing undergraduates at the start of their professional development. We used the original version here, obtaining results which showed consistent improvements among the four subscales, although subscale 4 had the lowest reliability. We believe that subscale 4 should be kept in the model, even for younger students, because it reflects an important aspect of interprofessional education related to understanding the contribution from all healthcare professions.

We found a significant modifying effect of gender as regards subscale 4: women had an empathic attitude towards understanding others’ values, whereas men did not. To the best of our knowledge, no previous study using the IEPS has shown such a significant gender effect. However, this result is consistent with other studies adopting different instruments. One is that of Wilhelmsson [28], in which a sample of 670 students were examined by the “Readiness for Interprofessional Learning Scale” to evaluate the results of IPE training, and showed that female students were more likely to work in a team. Another study by Hansson [29], using the “Jefferson Scale of Attitudes toward Physician-Nurse Collaboration” for medical and nursing students from two universities, showed a more positive attitude towards teamwork among female students.

In addition to gender, other characteristics regarding students' experience in the healthcare field were examined, such as having a physician and/or healthcare worker in the family. These features were assessed because students may begin their degree course already with a stereotyped vision about medical doctors or other healthcare professions. Such stereotyped perspective may alter their perception of the capability and skills of these professional figures [13, 30–32]*.*

Our results showed that students with a doctor or healthcare worker in the family did not show any improvement in “Perceived Need for Cooperation” or “Understanding Others' Values". Although our IPE training took place in the second year of medical school, before the start of clinical practice, it is however possible that some students came to university with already stereotyped attitudes towards other professions in the healthcare field [13, 30–32]. It has been suggested that these stereotypes are adopted more frequently among students with family members who work in healthcare. Tunstall-Pedoe [32], studying possible stereotypes in healthcare students following interdisciplinary courses, demonstrated that students with family members working in healthcare increased their likelihood of holding views which society accepts. Other student features, such as previous study, working or voluntary experience in the healthcare field, and previous history of hospitalisation did not modify our positive findings.

Our results clearly indicate that targeting one homogeneous group of students with IPE strategies based on real clinical experience and teaching by other health professionals can be effective and feasible with limited resources. In fact, no additional classes, seminars or teaching lessons were added to students' already busy calendar, and training was spread over one or two weeks, after reciprocal agreement between training ward personnel and students. However, as our study design did not include a control group, the results must be taken with a degree of scepticism. Our study reports on an interprofessional learning program targeted specifically on medical students who learn from and about other professions through interactions with qualified health professionals, and we did not find any directly comparable study in the reported literature. Another limitation to our study is that students’ attitudes may be influenced by other factors, over which we had no control. Beliefs and attitudes do not indicate true skills in interprofessional work. Therefore, one aspect to be further investigated is the ability to work as an active member of a multiprofessional team. Measurement of attitudes and skills by the IPE remains an open question, since no single instrument offers a sufficient solution to many educators and research teams [33]. We adopted only the IEPS to assess the effectiveness of our IPE training. Another widely used instrument which could have been included is the "Readiness for Interprofessional Learning Scale" (RIPLS) [34], developed to assess the attitudes and perceptions of students and professionals and to determine their readiness for interprofessional learning. Although our results are promising, the long-lasting effect of IPE training was not evaluated. Our results should be interpreted with caution, because students' socio-demographic characteristics and cultural backgrounds, as well as the stereotypes, expectations and attitudes which they bring to higher education, vary considerably between countries and institutions, and may influence IPE experience and learning [26]. This means that effective IPE activities in one university may not be as effective in another [35]. University IPE programs should provide more comparable methodologies and adopt pre-post evaluations more systematically.

## Conclusions

Our IPE training is innovative, in that it combines two different strategies - observation-based and practice-based learning. This may be viewed as effective, because the results indicate that students’ perception of IPC generally improved. However, interestingly, the results showed a lack of effectiveness of our educational strategies among students with a doctor or healthcare worker in their family, which did not change the perceived need for teamwork with other professionals or improve understanding of the other professionals’ values. More research is needed to explore associations between interprofessional education and healthcare teamwork and collaboration, including and comparing other scales for better understanding of improvements to interprofessional training outcomes and their long-lasting effects.

Our findings suggest that university-based IPE for medical students is feasible and effective. As a results, it seems desirable that it should be considered in all the core-curricula of healthcare professions, to foster positive attitudes to interprofessional collaboration in all future workers in this field.

## Additional file

Additional file 1:
**Frequency of 18 items in IEPS on medical students' pre-post training (n = 277).**

## Abbreviations

IPC: Interprofessional collaboration
IPE: Interprofessional education
IEPS: Interdisciplinary education perception scale
