# Supplementary material for: Effectiveness of interprofessional education by on-field training for medical students, with a pre-post design
Source: BMC Med Educ. 2015 Jul 29;15:121. doi: 10.1186/s12909-015-0409-z (PMC4518727; doi:10.1186/s12909-015-0409-z)
Supplement: Additional file 1: — Frequency of 18 items in IEPS on medical students' pre-post training (n = 277). [file 12909_2015_409_MOESM1_ESM.pdf]

Additional file 1 Frequency of 18 items in IEPS on medical students' pre-post training (n=277)

Items	Score	1	2	3	4	5
1.Individuals in my profession are well-trained.	Pre	1(0.36)	12(4.33)	42(15.16)	152(54.87)	70(25.27)
	Post	2(0.72)	6(2.17)	38(13.72)	131(47.29)	100(36.10)
2. Individuals in my profession are able to work closely with individuals in other professions .	Pre	0(0.00)	6(2.17)	74(36.71)	126(45.49)	71(25.63)
	Post	0(0.00)	2(0.72)	46(16.61)	152(54.87)	77(27.80)
3. Individuals in my profession demonstrate a great deal autonomy.	Pre	3(1.08)	24(8.66)	115(41.52)	110(39.71)	25(9.02)
	Post	0(0.00)	23(8.30)	82(29.60)	133(48.01)	39(14.08)
4.Individuals in other professions respect the work done by my profession.	Pre	0(0.00)	10(3.61)	73(26.35)	156(56.32)	38(25.27)
	Post	0(0.00)	6(2.17)	39(14.08)	165(59.57)	67(24.19)
5. Individuals in my profession are very positive about their goals and objectives.	Pre	0(0.00)	3(1.08)	58(20.94)	159(57.40)	57(20.58)
	Post	0(0.00)	1(0.36)	23(8.30)	161(58.12)	92(33.21)
6. Individuals in my profession must depend upon the work of people in other professions.	Pre	0(0.00)	1(0.36)	14(5.05)	88(31.77)	174(62.81)
	Post	1(0.36)	0(0.00)	4(1.44)	70(25.27)	202(72.92)
7. Individuals in my profession are very positive about their contributions and accomplishments.	Pre	0(0.00)	2(0.72)	24(8.66)	155(55.96)	96(36.66)
	Post	0(0.00)	1(0.36)	9(3.25)	147(53.07)	120(43.32)
8. Individuals in my profession must depend upon the work of people in other professions.	Pre	0(0.00)	14(5.05)	62(22.38)	125(45.13)	76(27.44)
	Post	0(0.00)	9(3.25)	44(15.88)	110(39.71)	114(41.15)
9. Individuals in other professions think highly of my profession .	Pre	6(2.17)	40(14.44)	164(59.20)	55(19.85)	12(4.33)
	Post	2(0.72)	31(11.19)	123(44.40)	106(38.27)	15(5.41)
10. Individuals in my profession trust each other’s professional judgment.	Pre	7(2.53)	33(11.91)	138(49.81)	91(32.85)	8(2.89)
	Post	2(0.72)	20(7.22)	111(40.07)	118(42.60)	26(9.39)
11.Individuals in my profession have a higher status than individuals in other professions.	Pre	22(7.94)	29(10.47)	73(26.53)	123(44.40)	30(10.83)
	Post	23(8.30)	43(15.52)	83(29.96)	98(35.38)	30(10.83)
12. Individuals in my profession make every effort to understand the capabilities and contributions with other professions .	Pre	5(1.80)	61(22.02)	145(52.35)	56(20.22)	10(3.61)
	Post	3(1.08)	48(17.32)	128(46.21)	81(29.24)	17(6.14)
13. Individuals in my profession are extremely competent.	Pre	1(0.36)	15(5.41)	145(52.35)	99(35.74)	17(6.14)
	Post	0(0.00)	3(1.08)	99(35.74)	141(50.90)	34(12.27)
14. Individuals in my profession are willing to share information and resources with other professionals.	Pre	0(0.00)	20(7.22)	133(48.01)	98(35.38)	26(9.39)
	Post	0(0.00)	12(4.33)	69(24.91)	138(49.82)	58(20.94)
15. Individuals in my profession have good relations with people in other professions.	Pre	1(0.36)	12(4.33)	146(52.71)	99(35.74)	19(6.86)
	Post	0(0.00)	10(3.61)	61(22.02)	148(53.43)	58(20.94)
16. Individuals in my profession think highly of other related professions.	Pre	3(1.08)	28(10.11)	143(51.62)	83(29.96)	20(7.22)
	Post	0(0.00)	12(4.33)	94(33.93)	136(49.10)	35(12.63)
17. Individuals in my profession work well with each other.	Pre	0(0.00)	14(5.05)	134(48.37)	109(39.35)	20(7.22)
	Post	2(0.72)	5(1.80)	67(24.19)	164(59.20)	39(14.08)
18. Individuals in my profession often seek the advice of people in my profession.	Pre	0(0.00)	16(5.78)	112(40.43)	124(44.76)	25(9.02)
	Post	0(0.00)	11(3.97)	70(25.27)	152(54.87)	44(15.88)
